# Alzheimer’s disease large-scale gene expression portrait identifies exercise as the top theoretical treatment

**DOI:** 10.1038/s41598-022-22179-z

**Published:** 2022-10-13

**Authors:** Mason A. Hill, Stephen C. Gammie

**Affiliations:** Department of Integrative Biology, University of Wisconsin-Madison, Madison, USA

**Keywords:** Alzheimer's disease, Neuroscience, Diseases of the nervous system

## Abstract

Alzheimer’s disease (AD) is a complex neurodegenerative disorder that affects multiple brain regions and is difficult to treat. In this study we used 22 AD large-scale gene expression datasets to identify a consistent underlying portrait of AD gene expression across multiple brain regions. Then we used the portrait as a platform for identifying treatments that could reverse AD dysregulated expression patterns. Enrichment of dysregulated AD genes included multiple processes, ranging from cell adhesion to CNS development. The three most dysregulated genes in the AD portrait were the inositol trisphosphate kinase, ITPKB (upregulated), the astrocyte specific intermediate filament protein, GFAP (upregulated), and the rho GTPase, RHOQ (upregulated). 41 of the top AD dysregulated genes were also identified in a recent human AD GWAS study, including PNOC, C4B, and BCL11A. 42 transcription factors were identified that were both dysregulated in AD and that in turn affect expression of other AD dysregulated genes. Male and female AD portraits were highly congruent. Out of over 250 treatments, three datasets for exercise or activity were identified as the top three theoretical treatments for AD via reversal of large-scale gene expression patterns. Exercise reversed expression patterns of hundreds of AD genes across multiple categories, including cytoskeleton, blood vessel development, mitochondrion, and interferon-stimulated related genes. Exercise also ranked as the best treatment across a majority of individual region-specific AD datasets and meta-analysis AD datasets. Fluoxetine also scored well and a theoretical combination of fluoxetine and exercise reversed 549 AD genes. Other positive treatments included curcumin. Comparisons of the AD portrait to a recent depression portrait revealed a high congruence of downregulated genes in both. Together, the AD portrait provides a new platform for understanding AD and identifying potential treatments for AD.

## Introduction

Alzheimer’s disease (AD) is the most common disease that causes dementia and can include the formation of neurofibrillary tangles of Tau proteins, accumulation of beta-amyloid plaques in major hubs of the brain, and cortical atrophy^1^. AD is a complex disorder that involves dysregulated expression of thousands of genes across multiple brain regions^2^ and is difficult to treat^3^. Large scale gene expression studies of post-mortem human AD and control brains have provided unique insights as these offer specific information on how gene expression patterns across ~ 20,000 protein coding genes are altered and in which directions in association with AD^2,4–7^. While a given brain region has a unique gene expression profile as do individual cells, common patterns of dysregulation across brain regions can be expected given similar pathologies are found across the CNS with AD^1^. Evaluation of common cross CNS dysregulation patterns is useful because it can identify a consistent signature of the disorder. Further, treatments or drugs can have a common signature of expression changes across regions. This study used signature matching within a drug repurposing framework where the goal is to find treatments that reverse expression patterns in a disorder^8–10^. This approach has been used successfully to identify new treatments for various disorders^11–13^, but a caveat is that for a given disorder it is not known whether expression reversal would lead to a treatment and the approach here is meant to be exploratory. In recent work, we created a portrait of depression that identified consistent patterns of dysregulated gene expression across regions and also evaluated how different treatments could reverse that pattern at the large-scale gene expression level^14^. In the present study, we use the same approach and include 22 datasets that were derived from 67 publicly available human AD gene expression datasets to create a gene expression portrait of AD that captures consistent patterns of gene expression dysregulation across brain regions. We also create separate AD portraits for men and for women to investigate possible differences in gene expression.

We first evaluate the expression portraits using enrichment analysis and explore the similarities and differences between the male and female AD portraits. We also evaluate the portrait with results from a recent AD GWAS study^15^ and identify dysregulated AD transcription factors that in turn affect transcription of other AD genes. We then use drug repurposing approaches to evaluate the AD portrait as well as the male and female AD portraits with over 250 potential treatment datasets. These include datasets for various drugs, each of which is also derived from large scale gene expression analysis of the CNS. Among the datasets are those for activity from humans that had been used previously to identify exercise as a possible AD treatment^16^ and for wheel-running from rodents. We also create a composite of exercise actions on the CNS from 11 human and rodent datasets and use this in analyses. Exercise was a focus of interest given multiple recent studies identifying the beneficial effects of exercise for mitigating AD-related deficits and how that may occur^16–20^. Together, we use multiple approaches, including hypergeometric analysis, heatmaps, and Uniform Manifold Approximation and Projection (UMAP)^21^ to identify the top theoretical treatments for reversing AD dysregulated gene expression patterns. The analysis also allows us to identify potential problematic treatments that may exacerbate AD gene expression patterns.

We compared the AD portrait approach described here with a portrait created using identical datasets and the MetaVolcano approach and with three other recent studies of AD using a meta-analysis approach^22–24^. Further, we compared the different meta-analysis datasets with each of the treatments. Given that there may be treatment effects that are region specific, we also compared 25 AD datasets that represent a range of CNS regions with each of the treatments. As a final step, we also compared the AD portrait with the previously published depression portrait^14^ to explore possible congruence between the disorders. Together, the goal of this study was to create a portrait of AD that identifies common dysregulated gene expression patterns in AD which may provide new insights into the disorder and to use the portrait in an exploratory manner for identifying how potential treatments, such as exercise, may affect AD at the specific gene expression level.

## Results

### Analysis of genetic findings in the portraits of AD

The top 1000 dysregulated genes in the AD portrait included 471 upregulated and 529 downregulated genes. The top dysregulated genes were the inositol-trisphosphate kinase, ITPKB (upregulated), the astrocyte-specific gene, GFAP (upregulated), the Rho GTPase gene, RHOQ (upregulated), the transcriptional repressor, NACC2 (upregulated), and the dystrophin-related gene DNTA (upregulated). In terms of common neurotransmitter systems and their receptors, the neuropeptides CRH, SST, PNOC, and VIP were all downregulated. Similarly, the enzymes involved in GABA synthesis, GAD1 and GAD2, were downregulated. The GABA receptors GABBR2, GABRA1, GABRB3, GABRG2 were downregulated. The glutamate receptors, GRIK1 and GRIK2 were also downregulated as was the SST receptor, SSTR1. The full list of the AD portrait with a ranking of all genes from most to least dysregulated with AD along with information on direction of expression change (up in AD = positive sign, down in AD = negative sign) is provided in Supplementary File 1.

Enrichment analysis (Toppcluster) of the top 1000 AD genes indicated enrichment for cell adhesion molecule binding, transporter activity, cytoskeletal protein binding, calmodulin binding, and actin binding. Additional enrichment was found for response to peptide hormone, protein localization to synapse, learning or memory, central nervous system development, cellular response to oxygen-containing compound, membrane fusion, trans-synaptic signaling, cell junction, protein phosphorylation, circulatory system development, regulation of programmed cell death, and mitochondrial membrane. BioPlanet2019 (within Enrichr) analysis also highlighted enrichment for BDNF signaling and MAPK signaling pathway. The BDNF gene that is part of both pathways is downregulated in AD. Supplementary File 1 provides enrichment analysis for ToppCluster and BioPlanet2019 (within Enrichr).

When comparing 642 GWAS genes associated with AD from a recent study^25^ to the top 1000 portrait genes, 41 common genes were identified and hypergeometric overlap of these two lists was significant (p < 0.0001). Some of the genes of interest included the neuropeptide, PNOC, the immune related gene, C4B (upregulated and also the 14th most dysregulated AD gene), the transcription factor, BCL11A (downregulated and also the 20th most dysregulated gene), and additional genes previously linked with AD, including ANK3, MS4A6A, AGFG2, CYC1, HLA-DRA, MEG3, MT2A, NCALD, NEU1, PSMC3, SERPINB6, and SPARC (see full list in Supplementary File 1).

**Figure 1:**
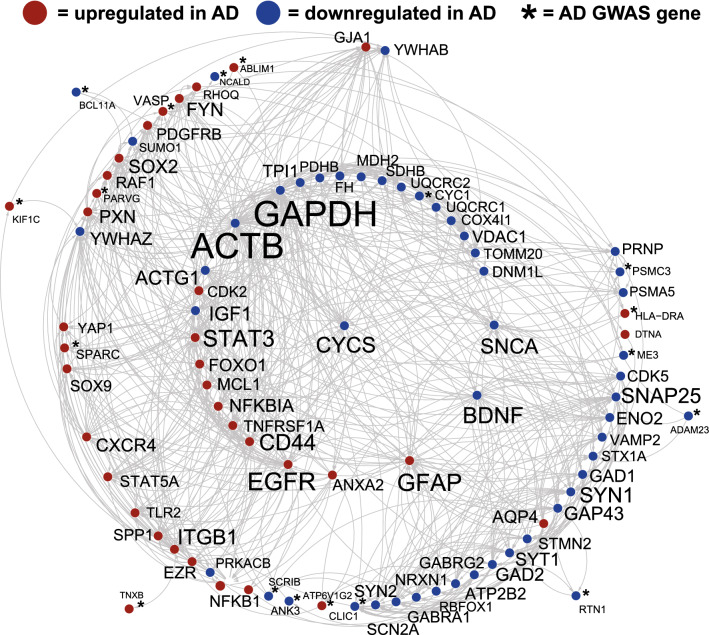
Top connected genes in AD portrait. Genes with the highest levels of protein–protein interaction (determined via STRING^15^) from the top 1000 dysregulated genes in AD portrait are plotted in Cystoscape^109^. Interactions are highlighted by lines. AD upregulated genes are shown in red and AD downregulated genes are show in blue. Increased size of font for gene symbol reflects higher number of connections between genes. GAPDH and ACTB were the two genes with the most connections. Also plotted are a subset of AD portrait genes also identified in a recent GWAS analysis of AD (shown with an asterisk). Steps for identifying top interacting genes are provided in the “Methods” section.

The protein interaction tool, STRING, was used to identify the portrait genes with the highest level of interaction with one another as this could provide insight into synergistic effects of altered expression (Fig. 1). Among the top connected genes were: ACTB, GAPDH, EGRF, SNAP25, STAT3, CYCS, SNCA, ACTG1, and BDNF. A subset of the portrait genes that were also in the AD GWAS study were also plotted and among these, ATP6V1G2, PSMC3, RTN1, NCALD, SPARC, ANK3, and SCRIB showed a high level of interaction with other genes (Fig. 1). When evaluating the top 5 dysregulated portrait genes, only GFAP was highly connected (Fig. 1). Enrichment for the plotted genes included: mitochondrion, cell junction, immune system, actin cytoskeleton, regulation of phosphorylation, and KEGG pathway AD genes, which included SNCA, GAPDH, and CYCS.

Using multiple transcription factor tools within EnrichR, 42 transcription factors were identified that were both within the top 1000 dysregulated AD genes and modify transcription of other AD dysregulated genes (top 1000 up and 1000 down) (Supplementary File 1). STAT3, SOX9, ELK1, and SOX2 were among the transcription factors with the highest level of evidence for altering other AD genes based on Enrichr matching using multiple datasets. Other transcription factors of interest included PAWR, GLIS3, AFF1, TCF3, FOXO1, BCL6, CEBPD, YAP1, RXRA, NFKB1, and NEUROD6 as these have previously been linked to AD, as detailed in the Discussion. Transcription factors altered in AD that in turn affect expression of other AD genes could have a large impact in the development of AD. Analysis of the 42 transcription factors indicated enrichment for nervous system development, cellular response to cytokine signaling, response to oxygen containing compounds, regulation of cell death, and signaling by interleukins.

**Figure 2:**
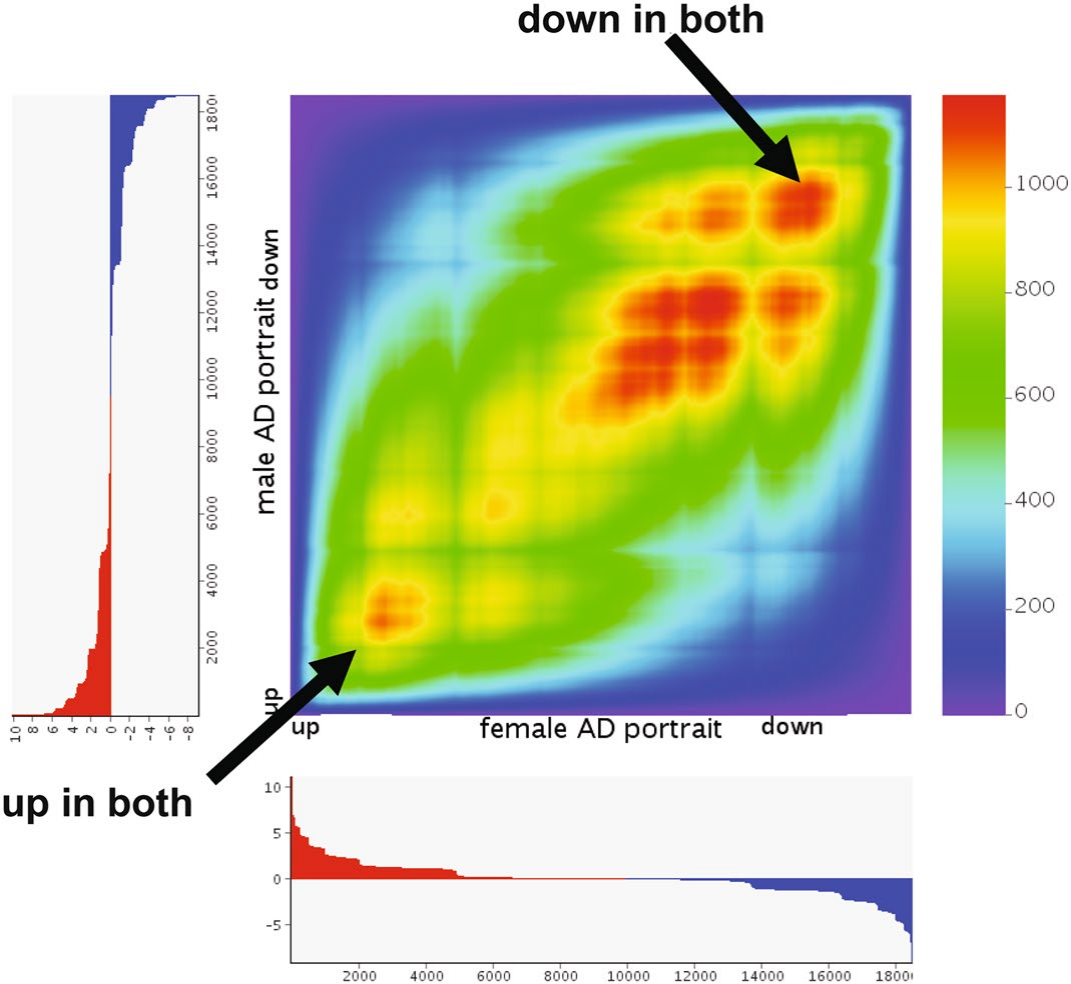
High congruence of male and female AD portraits. RRHO heat map^111^ is shown for comparisons of the male AD portrait (Y axis) and the female AD portait (X axis). Color is − log transformed hypergeometric p value showing the strength of the overlap as positive or negative enrichment. There is a high level of similarity in downregulated genes in both male and female AD datasets (red in upper right quadrant; see arrow) and in upregulated genes in both datasets (red in lower left hand quadrant; see arrow). All genes are used in RRHO analysis and upregulated genes are shown in red and downregulated genes shown in blue in the axis for each comparison.

When comparing the female and male AD portraits, there was a remarkably high congruence. 571 of the top 1000 upregulated genes in both were the same (hypergeometric p value = 0) and 570 of the top 1000 downregulated genes in both were the same (hypergeometric p value = 0). Further, not one gene was found to be ranked in the top 1000 in the opposite direction for the two groups (e.g., up in males, down in females). An RRHO heat map shows that the level of congruence between men and women with AD extends beyond the top 1000 genes (Fig. 2). In order to explore more subtle differences in AD between men and women, genes found in the top 500 in one group, but not in the top 1000 in the other were identified and then analyzed with STRING to identify top interacting genes. In females with AD, highly interacting genes included GAPDH, CYCS, SOX2, and PHGDH, while in males with AD the highly interacting genes included TLR2, ITGB2, NFKB1, and CD53.

### Analysis of AD portraits with treatments at the large-scale gene expression level

The AD portraits were used with drug repurposing approaches to evaluate potential treatments. The approach was to identify treatments that reverse large-scale gene expression dysregulation in AD, but do not worsen or push AD patterns in the same direction. Most treatment studies were from rodents. For the AD portrait, the top three scoring treatments for reversing AD expression with little effect on exacerbating AD expression were for exercise. A human CNS study comparing individuals with high versus low or high versus medium lifetime activity^16^ were the first and third top matches, respectively. An exercise composite that combined results from 11 exercise datasets, including those from human and rodents, was the second ranked treatment. In the top 25 were also two datasets examining exercise effects on the CNS in mice. Table 1 provides the top 25 treatments for each of the AD portraits. The full list of treatments along with their scores for reversing AD gene expression patterns for the three portraits (combined, female, and male) is provided in Supplementary File 2. The antidepressant, fluoxetine, ranked fourth and a composite of fluoxetine that included results from 13 fluoxetine treatment datasets ranked 13th. In the top 25, there were four fluoxetine datasets plus the fluoxetine composite. Curcumin, the plant chemical from Turmeric, was ranked fifth. Also in the top ten were desipramine and D-serine. Safflower oil (compared with flaxseed oil) in a high fat diet was the 6th highest treatment. The stimulant, cocaine, had three matches in the top 25. All three portraits had the same exercise dataset as the top ranked treatment and the exercise composite ranked third in both the female and male AD portraits. Overall, the ranking of treatments was similar for the three portraits, although in males, curcumin was the second highest ranked treatment (File 1, Supplementary File 2). In female and male AD portraits, the fluoxetine composite ranked 11th and 12th, respectively.

**Table 1:** Listing of the top 25 treatments for the AD portrait, the female AD portrait, and the male AD portrait.

Rank	GEO #	AD portrait		GEO #	Female AD		GEO #	Male AD	
		Treatment	Score		Treatment	Score		Treatment	Score
1	GSE110298	Exercise	121.46	GSE110298	Exercise	122.13	GSE110298	Exercise	81.63
2	11 Datasets	Exercise c*	68.70	GSE110298	Exercise	70.44	GSE33137	Curcumin	55.71
3	GSE110298	Exercise	63.05	11 Datasets	Exercise c*	70.06	11 datasets	Exercise c*	49.67
4	GSE84185	Fluoxetine	50.84	GSE10748	D-serine	52.07	GSE110298	Exercise	40.86
5	GSE33137	Curcumin	46.17	GSE10748	D-serine	51.46	GSE85936	Cocaine	37.49
6	GSE104338	Safflower oil	42.86	GSE27532	Desipramine	46.66	GSE84185	Fluoxetine	37.21
7	GSE27532	Desipramine	42.81	GSE104338	Safflower oil	40.77	GSE27532	Desipramine	35.84
8	GSE10748	D-serine	40.57	GSE84185	Fluoxetine	38.56	GSE10748	D-serine	25.55
9	GSE10748	D-serine	40.27	GSE33137	Curcumin	35.89	GSE74677	Chlorpyrifos	23.62
10	GSE84185	Fluoxetine	32.96	GSE7955	Permethrin	33.28	GSE10748	D-serine	23.03
11	GSE7955	Permethrin	31.24	13 Datasets	Fluoxetine c*	31.17	GSE104338	Safflower oil	22.37
12	GSE84185	Fluoxetine	28.62	GSE84185	Fluoxetine	31.09	13 Datasets	Fluoxetine c*	22.02
13	13 Datasets	Fluoxetine c*	27.14	GSE29075	Exercise	27.85	GSE84185	Fluoxetine	20.49
14	GSE29075	Exercise	26.77	GSE99349	Cocaine	23.13	GSE7955	Deltamethrin	20.11
15	GSE99349	Cocaine	25.93	GSE10748	D-serine	21.37	GSE7955	Permethrin	19.53
16	GSE85936	Cocaine	25.13	GSE84185	Fluoxetine	19.85	GSE99349	Cocaine	18.57
17	GSE64607	Exercise	24.95	GSE43748	Amphet.	19.76	GSE84185	Fluoxetine	18.50
18	GSE43748	Amphet.	24.49	GSE84185	Fluoxetine	18.69	GSE29075	Exercise	17.54
19	GSE74677	Chlorpyrifos	23.89	GSE7955	Deltamethrin	18.41	GSE64607	Exercise	17.29
20	GSE84185	Fluoxetine	22.25	GSE74677	Chlorpyrifos	18.21	GSE27532	Desipramine	16.63
21	GSE88736	Cocaine	20.89	GSE27532	Desipramine	17.68	GSE88736	Cocaine	15.82
22	GSE7955	Deltamethrin	20.82	GSE46717	Methamph.	16.70	GSE67755	Haloperidol	15.58
23	GSE10748	D-serine	18.06	GSE88736	Cocaine	16.58	GSE39980	Zonisamide	14.93
24	GSE81672	Imipramine	17.46	GSE64607	Exercise	16.50	GSE117758	Dexameth.	14.93
25	GSE27532	Desipramine	16.52	GSE9798	Corticost.	16.47	GSE81672	Imipramine	14.40

Additional details, including species, sex, and tissue source are provided in Supplementary Table S2.

c* composite, *corticost.* Corticosterone, *amphet.* amphetamine, *methamph.* methamphetamine, *dexameth.* dexamethasone.

The top ranked exercise dataset and the exercise composite were used to explore how exercise reversed AD gene expression patterns. The potential ability of exercise to reverse AD patterns was striking. For the top treatment, out of 1000 downregulated genes in AD, exercise reversed 225 (hypergeometric p value < 10^–74^) and out of 1000 upregulated AD genes, exercise reversed 184 (hypergeometric p value < 10^–48^). For the exercise composite out of 1000 downregulated genes in AD, exercise reversed 171 (hypergeometric p value < 10^–23^) and out of 1000 upregulated AD genes, exercise reversed 173 (hypergeometric p value < 10^–33^). Remarkably, very few genes moved in the same direction as in AD. For first and second ranked exercise treatments, only 20 and 45 AD genes moved in the same direction, respectively. Top cluster enrichment analyses of the 409 reversed AD genes by the top exercise treatment are shown in Supplementary File 2 and include: cell adhesion molecule binding, cytoskeletal protein binding, blood vessel morphogenesis, circulatory system development, blood vessel development, and neuron projection morphogenesis. One PubMed enrichment was for a protein-interaction network that interacts with interferon-stimulated genes. BioPlanet 2019 enrichment also highlighted N-cadherin signaling and neurotrophin signaling. For the exercise composite, Toppcluster enrichment analyses of the 344 reversed AD genes are shown in Supplementary File 2 and include: transcription factor binding, actin binding, synapse organization, cell junction organization, and brain development. PubMed enrichment for a protein-interaction network that interacts with interferon-stimulated genes was also found. BioPlanet 2019 enrichment also highlighted BDNF signaling pathway. The exercise composite is provided in Supplementary File 2.

**Figure 3:**
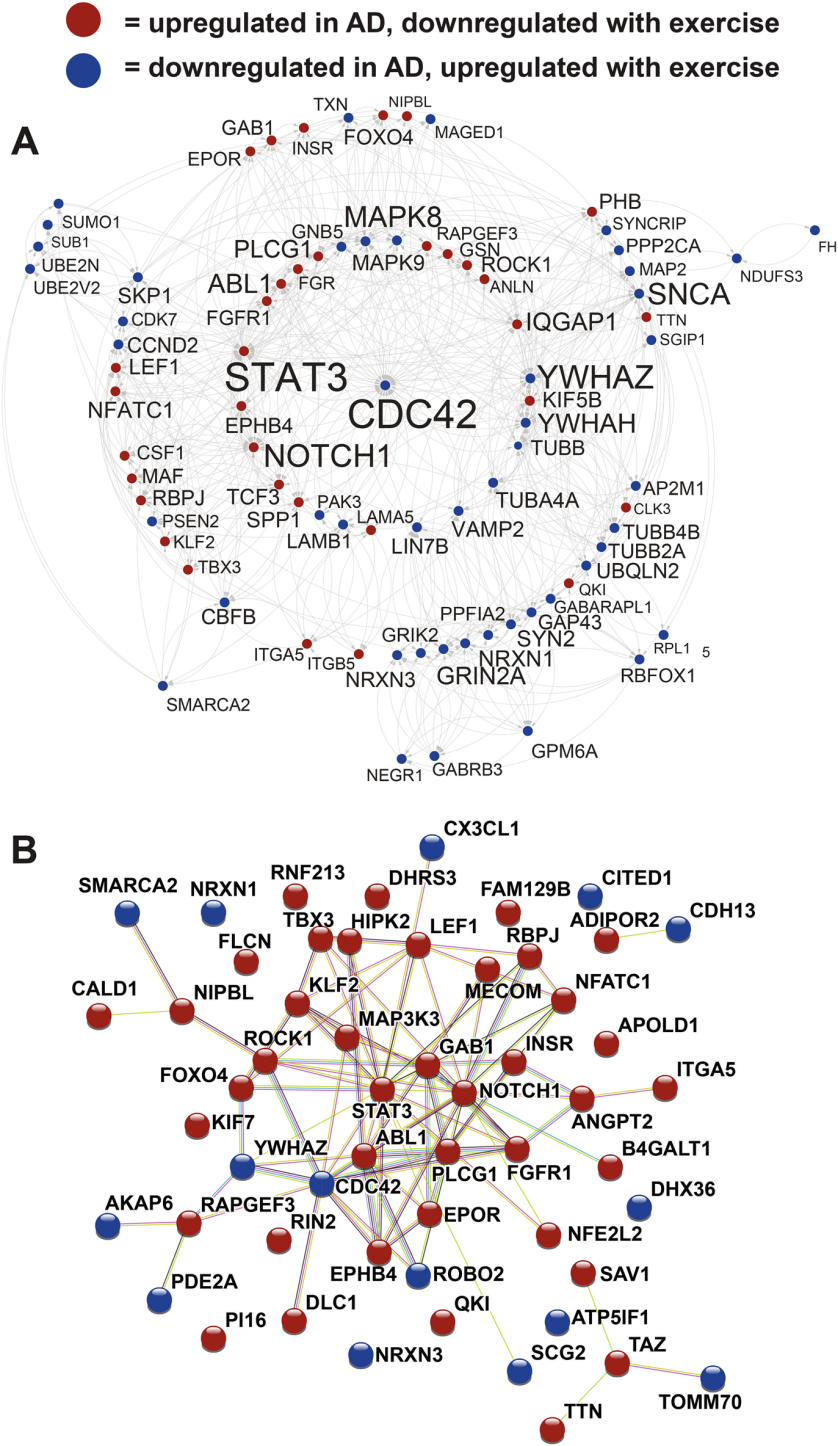
AD dysregulated genes reversed by exercise (human dataset). (**A**) Genes with the highest levels of protein–protein interaction (determined via STRING^15^) from the 409 AD genes reversed by exercise (top treatment; human high versus low activity) are plotted in Cytoscape^109^. Increased size of font for gene symbol reflects higher number of connections between genes. CDC42, STAT3, NOTCH1, SNCA, YWHAZ, and MAPK8 were among the genes with the most connections. Interactions are highlighted by lines. (**B**) Blood vessel development genes were enriched within the AD genes reversed by exercise and are plotted in STRING^15^. Connections between proteins are shown by lines and color of line indicates type of evidence: light blue (known interactions from curated databases); pink (known interactions that are experimentally determined); green (predicted interactions from gene neighborhood); red (predicted interactions from gene fusions); blue (predicted interactions from gene co-occurrence); light green (text mining); black (co-expression); and gray (protein homology). AD upregulated genes downregulated by exercise are shown in red and AD downregulated genes upregulated by exercise are shown in blue for both (**A,B**).

**Figure 4:**
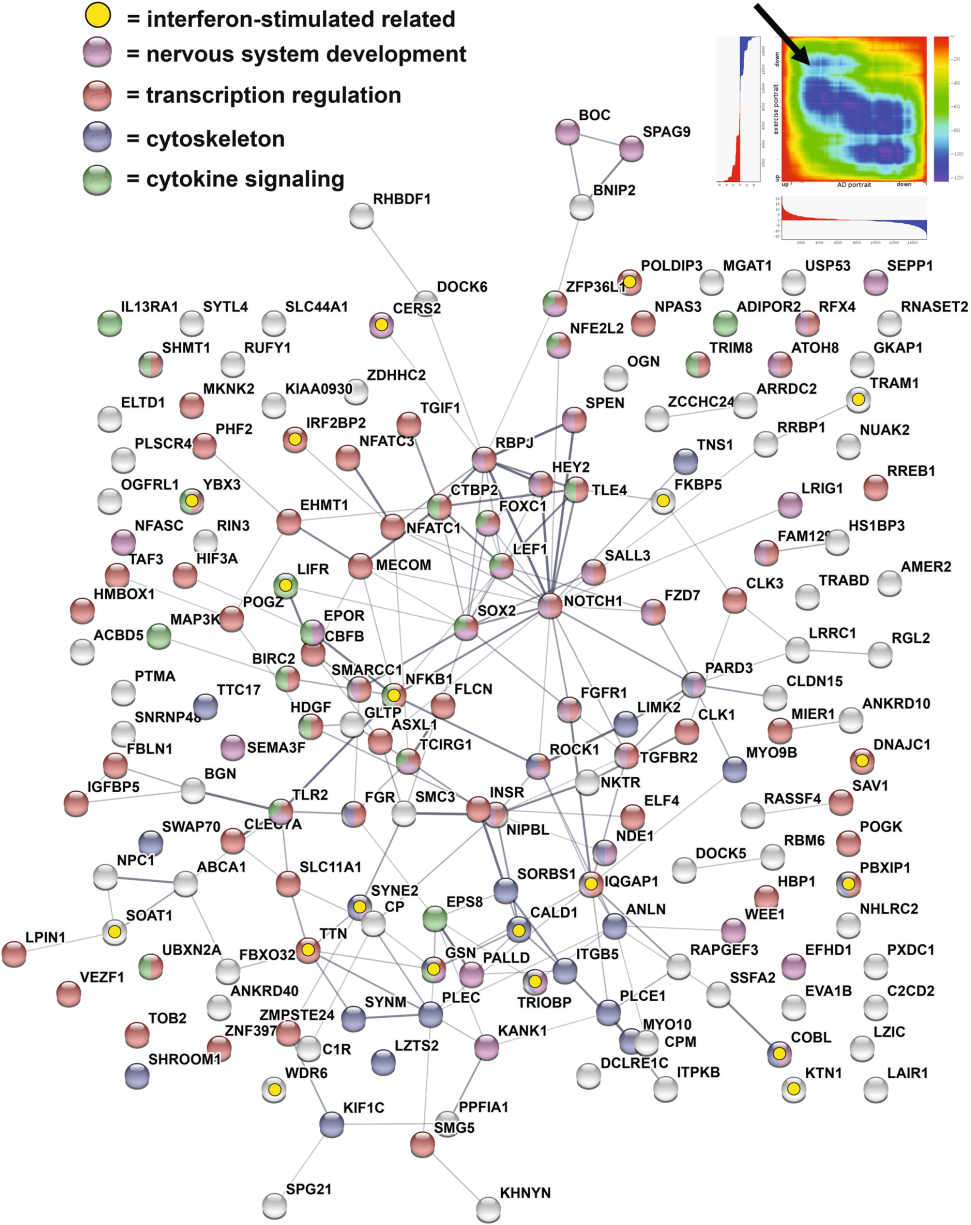
Genes upregulated in AD and downregulated with exercise composite. The 173 genes upregulated with AD and reversed by the exercise composite are plotted in STRING^15^. Lines between genes indicates connections and width of line reflects strength of evidence. Enrichment was found for: nervous system development (purple); transcription regulation (red); cytoskeleton (blue); cytokine signaling (green); and interferon-stimulated related (yellow). Upper right shows RRHO heat map^111^ comparing exercise composite (Y axis) and AD portrait (X axis). Arrow highlights reversal of upregulated genes in AD by exercise.

**Figure 5:**
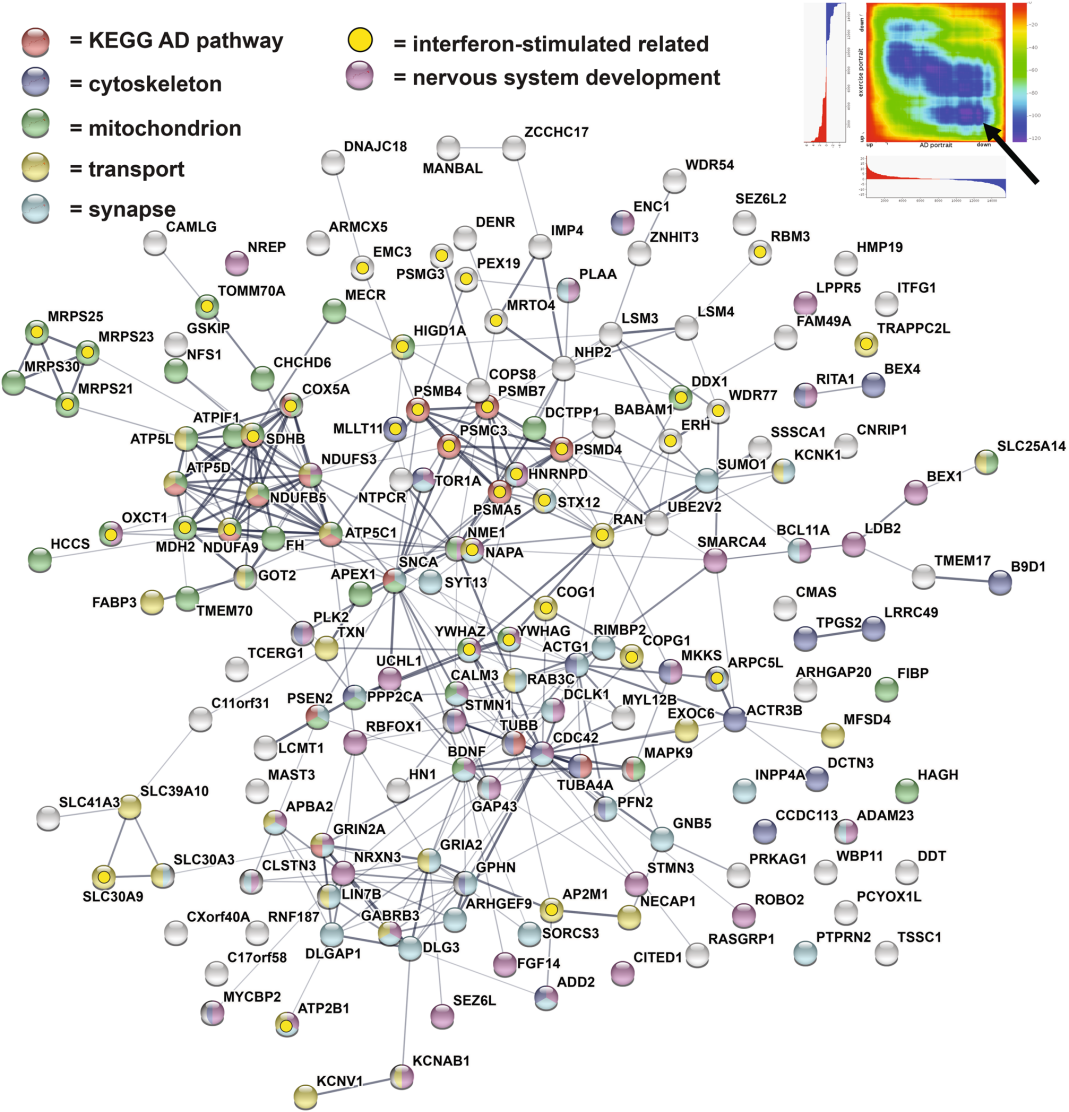
Genes downregulated in AD and upregulated with exercise composite. The 171 genes downregulated with AD and reversed by the exercise composite are plotted in STRING^15^. Lines between genes indicates connections and width of line reflects strength of evidence. Enrichment was found for: nervous system development (purple); KEGG AD pathway (red); cytoskeleton (blue); synapse (light blue); mitochondrion (green); cytoskeleton (light yellow); and interferon-stimulated related (yellow). Upper right shows RRHO heat map^111^ comparing exercise composite (Y axis) and AD portrait (X axis). Arrow highlights reversal of downregulated genes in AD by exercise.

STRING analysis of the 409 AD genes reversed by the top exercise dataset indicated a group of 82 genes with high interactions with one another and these are plotted in Cytoscape (Fig. 3A). Among the most connected genes were: CDC42, STAT3, NOTCH1, YWHAZ, SNCA, MAPK8, and ABL1. Enrichment for the highly connected genes included blood vessel development, PI3K-Akt signaling, and AD KEGG pathway. Genes related to blood vessel development that are dysregulated in AD and reversed by exercise are shown in Fig. 3B. For the exercise composite, reversal of genes upregulated in AD are shown in Fig. 4 and reversal of genes downregulated in AD are shown in Fig. 5. For reversed upregulated AD genes enrichment was found for nervous system development, interferon-stimulated related, transcription, cytoskeleton, and cytokine signaling (Fig. 4). For reversed downregulated AD genes enrichment was found for KEGG AD pathway, cytoskeleton, mitochondrion, transport, synapse, and nervous system development (Fig. 5). AD KEGG genes included: SNCA, GRIN2A, PSEN2, and MAPK9. ITPKB, the top dysregulated gene in the AD portrait, was reversed by the exercise composite as were five other top 20 AD dysregulated genes: ITGB5, PALLD, YWHAZ, TNS1, and BCL11A. BDNF is downregulated in AD, but upregulated by exercise. A list of AD genes reversed by exercise and that are moved in the same direction for the exercise composite and the top human and rodent exercise dataset and are provided in Supplementary File 2. A Rank Rank Hypergeometric Overlap (RRHO) heatmap reflects the reversal of exercise on up and down dysregulated genes in AD and this extends beyond the top 1000 upregulated and 1000 downregulated genes (Figs. 4, 5).

**Figure 6:**
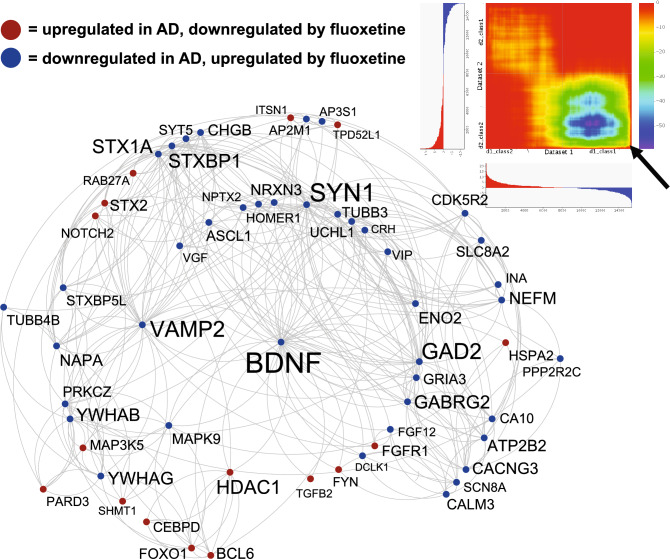
AD dysregulated gene expression reversed by fluoxetine composite. Genes with the highest levels of protein–protein interaction (determined via STRING^15^) from the 271 AD genes reversed by fluoxetine composite are plotted in Cytoscape^109^. Increased size of font for gene symbol reflects higher number of connections between genes. BDNF, SYN1, GAD2, and VAMP2 were among the genes with the most connections. Interactions are highlighted by lines. AD upregulated genes downregulated by fluoxetine are shown in red and AD downregulated genes upregulated by fluoxetine are shown in blue. Upper right shows RRHO heat map^111^ comparing fluoxetine composite (Y axis) and AD portrait (X axis). Arrow highlights reversal of downregulated genes in AD by fluoxetine.

For the fluoxetine composite (from 13 datasets), out of 1000 downregulated genes in AD, fluoxetine reversed 149 (hypergeometric p value < 10^–22^) and out of 1000 upregulated AD genes, fluoxetine reversed 100 (hypergeometric p value < 10^–05^). Of the 249 reversed genes, highly interactive proteins were identified in STRING and plotted in Cytoscape (Fig. 6). Top interacting genes reversed by fluoxetine included BDNF, SYN1, VAMP2, GAD2, STX1A, STXBP1, and HDAC1. Enrichment included nervous system development, response to stress, KEGG AD pathway, MAP kinase signaling, and synapse. When examining AD genes reversed by both fluoxetine and exercise composites, 44 genes were common, including BDNF. In a theoretical combining of fluoxetine and exercise, 549 AD genes would be reversed.

The scoring and ranking approach allowed for identification of treatments that could worsen AD and the top two datasets were from human CNS tissue and related to alcohol abuse (Supplementary File 2), suggesting alcohol at certain levels could be a risk factor for AD.

**Figure 7:**
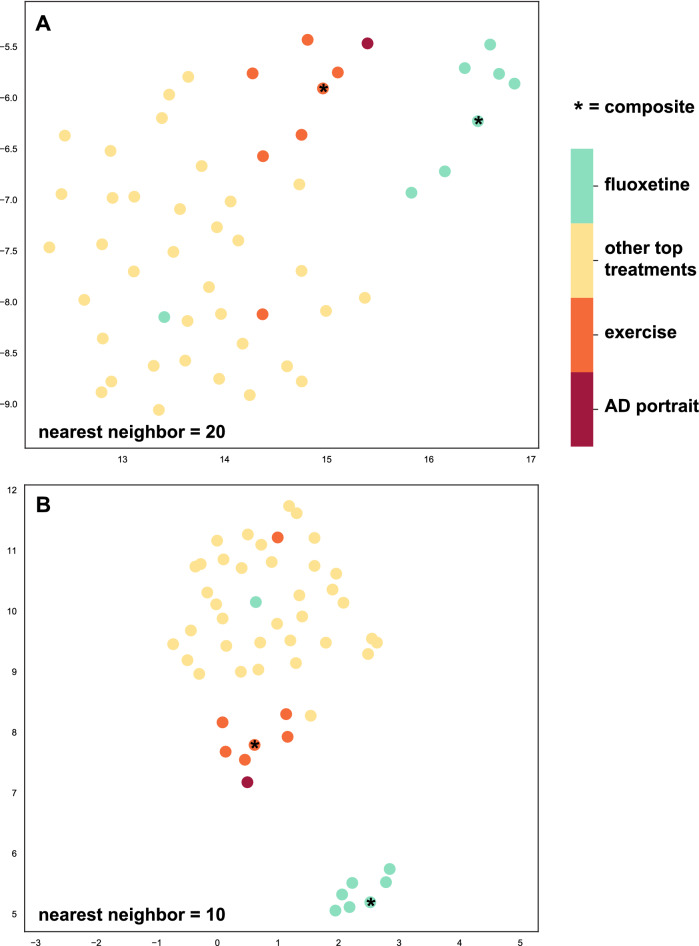
UMAP plotting of treatments with AD portrait. UMAP was used to incorporate data from complex landscape of multidimensional features (5862 genes) and flatten those to two dimensions to identify similarities between datasets. The sign for the AD portrait was reversed so that a closer distance (spatial proximity) of a treatment (top 51 plotted) to the portrait represents a better match (reversal of pattern). Plot represents higher dimension data plotted into two dimensions. The x‐ and y‐axes are arbitrary embedding dimensions generated by UMAP. Top graph (**A**) is from plotting using the neighborhood setting of 20 and lower graph (**B**) uses the setting of 10. The AD portrait was most closely associated with the exercise datasets, but was also close to the fluoxetine treatments. Colored dots are: dark red (AD portrait); orange (exercise); tan (other top treatments); turquoise (fluoxetine). The fluoxetine and exercise composites are denoted with an asterisk.

UMAP incorporates the complex landscape of multidimensional features, such as gene expression, and was used as an alternative approach to gain insights into which treatments may best reverse AD expression patterns. The sign for the AD portrait was reversed so that treatments that better reverse gene expression in AD will be plotted spatially closer to the reversed AD portrait. As shown in Fig. 7, when examining almost 6000 genes at once, exercise datasets, including the exercise composite, were most closely plotted with AD when using two different settings. Fluoxetine datasets, including the fluoxetine composite, clustered to together and were separate from other treatments, but were not as closely plotted to the portrait as was exercise.

### Comparisons with an alternative portrait approach and prior meta-analysis AD studies

The AD portrait showed a high level of matching when compared with an AD portrait made using the identical datasets and the MetaVolcano approach^26^ and two other recent meta-analysis studies^23,24^. It also matched a third meta-analysis study^22^, but at a lower levels. Supplementary File 3 contains the MetaVolcano AD portrait, its comparison to the individual AD datasets, and the comparisons of the different meta-analyses studies with one another.

### Comparison of meta-analysis approaches and individual region-specific AD datasets with treatments

When the MetaVolcano AD portrait and the three recent meta-analysis AD datasets were also tested with treatments, exercise was the top treatment for each (Supplementary File 4). Additionally, for the two GEO independent studies that had the highest number of samples (and were from prefrontal cortex and visual cortex), exercise and the exercise composite ranked first and second for both men and women with AD. High ranking of exercise was found for most of the individual datasets and region to region comparison can be made with treatments when available. For example, for 13 of the 25 individual AD datasets, exercise ranked as the first and second best treatment. For another 8 datasets, one or more exercise treatments were in the top 10. There was no clear pattern where tissue source from AD affected matching to treatments. For example, the best match to exercise (from hippocampus) was from an AD study in medial temporal cortex. Data from the Religious Order Study and the Memory and Aging Project (ROSMAP)^27,28^, Mayo clinic^29^, and Mount Sinai Brain Bank (MSBB)^30^ studies had been harmonized^31^ and were also compared with treatments (one region each). While each study highlighted exercise as a top 2 treatment, acetyl-l-carnitine as well as coral calcium hydride were also among the highest scoring treatments. Finally, the AD portrait was modified to include six datasets from the ROSMAP, Mayo, and MSBB harmonized differential expression analysis^31^. The top result of that modified portrait was exercise and the full list of genes in the modified portrait is also provided (Supplementary File 4). Full results on scoring and ranking of treatments with the respective studies are provided in Supplementary File 4.

### Comparisons of the AD and depression portrait

**Figure 8:**
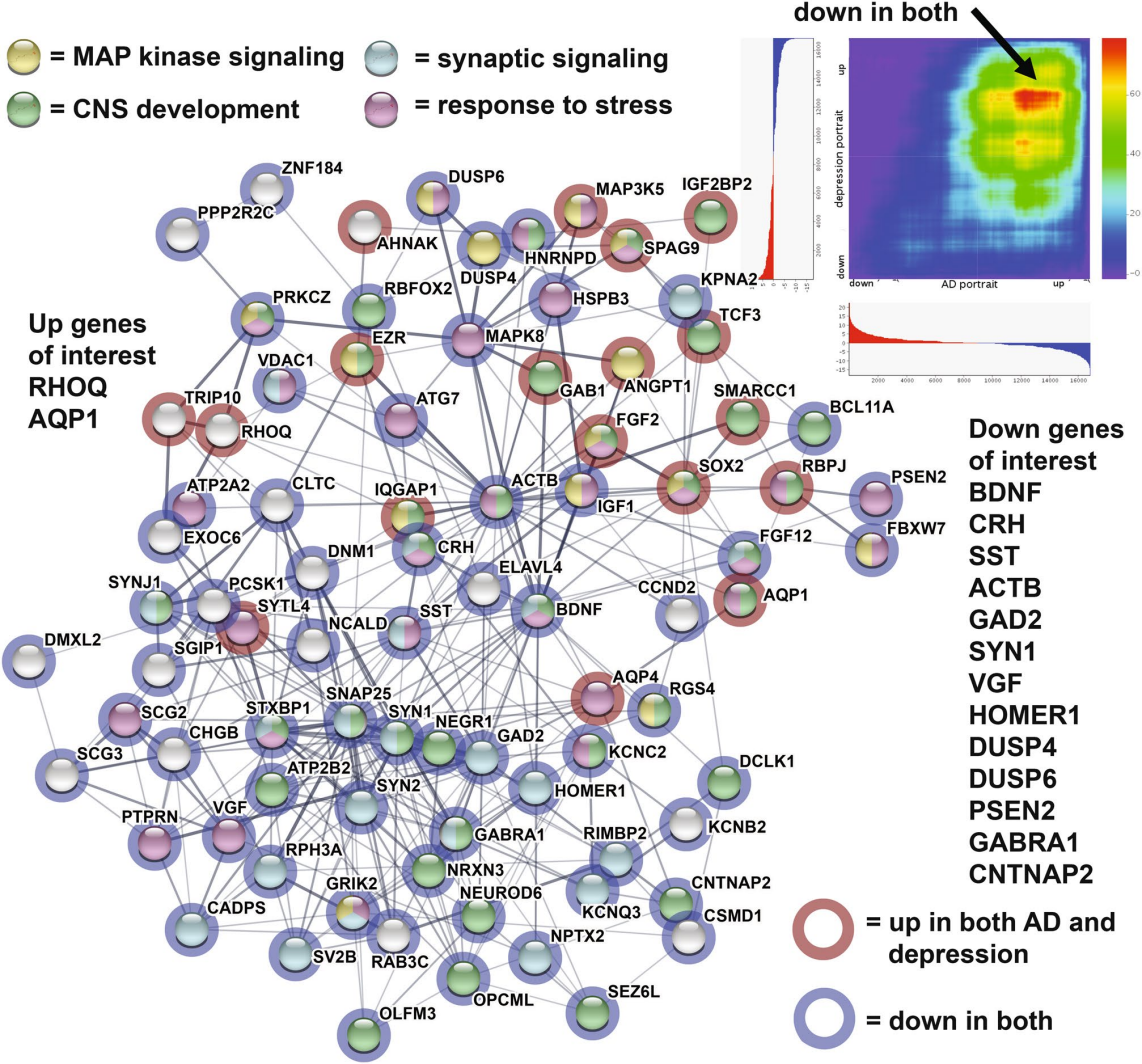
Congruence between AD portrait and depression portrait. Genes with the highest levels of protein–protein interaction (determined via STRING) from the 226 AD genes that match direction in both AD and depression are plotted in STRING^15^. Enrichment was found for: MAP kinase signaling (yellow); CNS development (green); synaptic signaling (light blue); and response to stress (purple). A red halo indicates genes upregulated in AD and depression and a blue halo indicates genes downregulated in both. Common genes of interest include: BDNF, CRH, SST, ACTB, GAD2, and PSEN2. Lines between genes indicates connections and width of line reflects strength of evidence. Upper right shows RRHO heat map^111^ for comparisons between depression (Y axis) and AD (X axis) and arrow highlight high congruence of common genes downregulated in both. Details on the axes and colors are same as in Fig. 2.

The AD portrait was compared with a recent portrait of depression^14^ as comorbidity of depression and AD occurs in some individuals. Out of 1000 downregulated genes in AD, 139 of the same genes were also down in the depression portrait (hypergeometric p value < 10^–21^) and out of 1000 upregulated AD genes, 87 of the same genes were up in depression (hypergeometric p value < 0.0001). Of these common AD and depression genes, a subset of highly connected genes are shown in Fig. 8. Some of the noteworthy downregulated genes in both and included: BDNF, CRH, SST, GAD2, PSEN2, DUSP4, DUSP6, HOMER1, VGF, ACTB, and GABRA1.

## Discussion

The AD portrait provides an overview of consistent gene expression dysregulation with AD across multiple brain regions and fits within the framework that similar pathologies are found across the CNS with AD^1^. An advantage of the AD portrait is that it allows for insights into global patterns, although it is less likely to reveal AD alterations that are specific to a region. However, given that many treatments have similar effects across brain regions, the portrait provides a platform for evaluating treatments, such as exercise, that could reverse the global patterns. As discussed below, the results of the AD portrait with treatments matches well those from individual AD datasets from specific regions with treatments, suggesting the portrait provides a robust platform for evaluating AD and potential treatments.

The top dysregulated gene was inositol-trisphosphate kinase, ITPKB (upregulated), that phosphorylates and converts the second messenger IP3 to IP4 and has been found to localize with actin^32^ and with amyloid plaques in human postmortem AD tissue^33^. While ITPKB is not commonly listed as being in the pathway for AD, its ability to affect IP3 levels that in turn influence intracellular calcium could connect it to the disease. Recent studies that support overexpression of ITPKB as causative of AD found in animal models that elevated ITBKB is linked to apoptosis and increased amyloid peptide production^34^ as well as TAU pathologies^33,35^. However, other studies suggest elevated ITPKB could be helpful in counteracting high Ca++ in mitochondria in neurologic pathologies^36^. Other inositol related genes dysregulated with AD were also upregulated, including ITPRID2, ITPRID, and ITPRIPL2A. The phospholipase C gene, PLCE1, that could affect IP3 levels was also upregulated. An understanding of whether or how elevated ITPKB contributes to AD remains to be determined. Interestingly, exercise reverses this overexpression pattern as discussed below.

The second most dysregulated gene was the astrocyte-specific gene, GFAP (upregulated), that is upregulated during inflammation and reactive gliosis, including within AD^37–39^. Other injury related genes were BDNF, AQP4, GAP43, GJA1, SOX9, CDK2, and EGFR. Overexpression of GFAP as occurs in Alexander's disease due to GFAP mutations, can lead to AD-like pathologies^40^. While early elevations of GFAP are expected as neuroprotective, prolonged overexpression could contribute to AD pathologies.

The Rho GTPase gene, RHOQ, was the third most dysregulated gene (upregulated) and it plays a role in actin cytoskeleton assembly^41^. Actin related processes were highly enriched among the top dysregulated AD genes. The transcription factor, NACC2 (upregulated), was the fourth most dysregulated gene and expression changes have been connected to Lewy bodies^42^. The dystrophin-related gene, DNTA (upregulated), was fifth ranked and DNTA localizes with the perivascular astrocytic endfoot and elevated levels are associated with increased AD pathologies^43^. Neuritin 1 (NRN1; downregulated) is involved in neuronal plasticity and associated with neurofibrillary tangles^44^ and was the sixth most altered gene in AD. The G protein regulating gene, RGS7 (downregulated) ranked as the seventh most disrupted gene and has been linked to multiple neuronal disorders^45^. While the majority of dysregulated genes are likely to have a contributory effect on AD, in future studies it will be important to evaluate which gene patterns are the most causative and which (if any) may seem like a dysregulation but are neuroprotective.

41 genes from a recent human GWAS AD study^25^ were among the top 1000 AD dysregulated genes (Fig. 1, Supplementary File 1), including the opioid neuropeptide, PNOC, downregulated and connected to AD^46^, the immune related gene, C4B, upregulated and linked with AD^47^, and the transcription factor, BCL11A, downregulated and associated with cortical neuron differentiation^48^. Additional GWAS genes previously linked with AD included: ANK3, MS4A6A, AGFG2, CYC1, HLA-DRA, MEG3, MT2A, NCALD, NEU1, PSMC3, SERPINB6, and SPARC (see full list in Supplementary File 1). How GWAS AD genes interact with or affect transcription of dysregulated AD genes remains to be determined.

In terms of common neurotransmitter pathways, the neuropeptides CRH, PNOC, VIP and SST (and its receptor SSTR1) were all downregulated as were the enzymes involves in GABA synthesis, GAD1 and GAD2. Potential roles for these signaling molecules in AD have been suggested^46,49–51^. Further, the GABA receptors GABBR2, GABRA1, GABRB3, GABRG2 and the glutamate receptors, GRIK1 and GRIK2 were also downregulated. While these neural signaling pathways were downregulated in AD, others were not suggesting potential specific roles for these signaling molecules in AD. As an example, elevation of GABA signaling has been proposed as a route for treatment of AD^52^.

Genes with the highest connections to one another (per STRING) within the top 1000 AD dysregulated genes included: ACTB, GAPDH, EGRF, SNAP25, STAT3, CYCS, SNCA, ACTG1, and BDNF (Fig. 1). It is possible that highly connected genes have an oversized effect on the AD phenotype given synergistic actions, but this is speculative. SNCA, GAPDH, and CYCS are part of the KEGG AD pathway^53^ and SNCA plays a role in the development of amyloid plaques^54^. Enrichment for the highly connected genes included mitochondrion, cell junction, immune system, actin cytoskeleton, and regulation of phosphorylation which is consistent with AD pathologies. BDNF is down regulated in the AD portrait and is part of both the BDNF and MAPK signaling pathways.

Forty-two transcription factors were identified with the top 1000 dysregulated AD genes also modified expression of other AD dysregulated genes (Supplementary File 2). STAT3, SOX9, ELK1, and SOX2 affected the most genes, but other transcription factors of interest included PAWR, GLIS3, AFF1, TCF3, FOXO1, BCL6, CEBPD, YAP1, RXRA, NFKB1, and NEUROD6 as these have previously been linked to AD within curated databases of diseases^53,55–57^. Transcription factors altered in AD that in turn affect expression of other AD genes could have a large effect on AD pathologies. Upregulated STAT3 contributes to astrogliosis and reversal of this pattern can mitigate AD phenotypes^58^. Similarly, SOX9 is elevated with AD and decreases in expression can reverse some AD markers^59,60^.

The female and male AD portraits were highly congruent with one another with 571 and 570 common genes the top 1000 up and downregulated genes, respectively. Further, no top 1000 gene was in the opposite direction. The RRHO heat map in Fig. 2 highlights the high congruence across levels of dysregulation. When focusing on the most dysregulated genes within each sex that were not found in the other sex, there were differences in highly interacting genes whereby in females GAPDH, CYCS, SOX2, and PHGDH had strong interactions, but in males TLR2, ITGB2, NFKB1, and CD53 interacted the most strongly. Our findings are similar to a recent study combining multiple datasets that also found significant overlap between males and females with AD when examining differential expression^22^. However, that study also found some sex differences in enrichment pathways and more extensive sex differences with AD when using Weighted Gene Co-expression Network Analysis (WGCNA)^61^ to identify gene networks. Our findings may differ for a few reasons, including that we did not systematically explore enrichment differences, we did not use WGCNA, and the results are based on different datasets. Given that the male AD portrait matched at high level the individual female AD datasets used to create the female AD portrait (and vice versa) (Supplementary File 1), we do not think our approach masked sex differences, but we cannot exclude this possibility.

Out of over 250 treatment datasets, the top three treatments were for exercise. A human CNS study comparing hippocampal gene expression in individuals with high versus low or high versus medium lifetime activity^16^ were the first and third top matches, respectively. The activity study also included a comparison of exercise modulated and AD modulated genes^16^ and identified some of the same genes of interest as in this study. The present approach differs in a few ways including that here multiple exercise/activity datasets were used, an exercise composite was created and used, and exercise was one of many possible treatments analyzed. The exercise composite that combined results from 11 exercise datasets, including those from human and rodents, was the second ranked treatment. In the top 20 were also two datasets examining exercise effects on the CNS in mice. Using UMAP as an alternative approach to identify the best treatments, exercise again stood out as a promising treatment (Fig. 7). These findings are consistent with a multitude of studies suggesting exercise in humans provides neuroprotective effects against development and progression of AD or related pathologies^17–20,62–64^.

The potential ability of exercise to reverse AD patterns was striking. For the first and second ranked treatments 409 and 344 AD genes were reversed while only 20 and 45 genes were in the same direction, respectively. Enrichment for AD genes reversed by the top exercise treatment included cell adhesion, cytoskeletal binding, neuron projection as well as multiple entries related to blood vessels, including blood vessel morphogenesis, circulatory system development, blood vessel development (Fig. 3B). These latter categories are of interest as decreased blood flow is associated with AD^65^ and exercise is posited to elevate brain blood flow as part of its effect on cognition^66^.

For the exercise composite (2nd ranked) enrichment of reversed AD genes included: transcription factor binding, actin binding, synapse organization, cell junction organization, brain development, BDNF signaling pathway, and a gene set from a recent study identifying interferon-stimulated network that is relevant to the innate immune response^67^. Previous studies have shown a link between interferon and exercise^68^ as well as possible roles for the innate immune system in the development of AD^69^. Whether or how exercise may invoke aspects of the innate immune response in the reversal of AD genes remains to be elucidated.

CDC42 (down in AD) is of interest as it had the greatest number of interactions with other genes reversed by exercise for the top two exercise datasets (Figs. 3, 5). CDC42 is a small GTPase of the Rho-subfamily and is connected to multiple pathways relevant to AD, including MAPK signaling, actin organization, cell junction, and CNS development. The possible role of CDC42 in AD may be complex as one line of research suggests inhibition as a pathway for treatment^70^, while another suggests activation as an approach to offset AD-like pathologies^71^. BDNF (down in AD) is reversed by the exercise composite and BDNF is well studied in terms of how it is upregulated by exercise and positively affects CNS function^72,73^ while its role in AD is still being explored^74,75^. Other genes reversed by exercise that are in the BDNF pathway include CDC42, NFKB1, MAPK8, and MAPK9. Twenty-two of the AD genes reversed by the exercise composite are part of the KEGG AD pathway, including SNCA, PSEN2, CALM3, GRIN2A, NFKB1, INSR, and TUBB. As indicated above, the top AD portrait dysregulated gene, ITPKB, is reversed by exercise. Together, exercise reverses a wide range of genes involved in a number of important AD-related processes. RRHO heat map analysis suggested exercise would also advantageously affect genes outside of the top 1000 dysregulated portrait genes (Figs. 4, 5).

The male and female portrait both had the same human exercise dataset as the top ranked treatment and the exercise composite ranked third in both. Further, the MetaVocano AD portrait as well as three recent meta-analysis datasets^22–24^ also had exercise as the top two treatments. While other AD meta-analysis studies exist^76–78^, full lists of genes were not provided so comparisons to treatments were not made. Importantly, exercise was also top ranked for both males and females within the two GEO datasets (GSE33000 and GSE44771)^6,7^ that were produced using the highest number of AD and control samples and came from prefrontal cortex and visual cortex, respectively. Results for 25 AD datasets that are brain region specific with each of the treatments are provided in Supplementary File 4. Exercise was highly ranked across the individual AD datasets with exercise ranked as the first and second best treatment for 13 of the 25 datasets. For another 8 datasets, one or more exercise treatments were in the top 10. There was not a clear pattern where tissue source affected matching to treatments and the best match to exercise (from hippocampus) was from an AD study in medial temporal cortex. The ROSMAP, Mayo, and MSBB studies each had exercise as a first or second top treatment, but interestingly there were high rankings for acetyl-L-carnitine which has been investigated for possible AD-related treatments^79,80^. In summary, the high ranking of exercise was robust across meta-analysis studies, across the individual datasets with the highest number of samples, and across most of the other AD datasets.

The antidepressant, fluoxetine, ranked fourth and a composite of fluoxetine that included results from 13 fluoxetine treatment datasets ranked 13th. In the top 25, there were four fluoxetine datasets plus the fluoxetine composite. Some of the top connected AD genes reversed by fluoxetine included BDNF, SYN1, VAMP2, GAD2, STX1A, STXBP1, and HDAC1 (Fig. 6). In both female and male AD portraits, the fluoxetine composite ranked 11th and 12th, respectively. This finding is consistent with recent work in animals and humans that fluoxetine can be a useful treatment for AD-related conditions^81–83^. When examining AD genes reversed by both fluoxetine and exercise composites, 44 genes were common, including BDNF, and a theoretical combining of the two treatments would reverse 549 AD genes. This finding supports recent work exploring the combination of both exercise and fluoxetine for AD and other disorders^84^.

Curcumin, the plant chemical from Turmeric, acting in cortex was ranked fifth and this is consistent with studies examining the therapeutic effects of curcumin in the treatment of AD^85–87^. However, curcumin acting in hippocampus had a slightly negative effect and datapoints from different ages had to be combined to achieve sufficient numbers. To our knowledge, a study examining curcumin effects in the CNS with a high number in each group has yet to be performed. Desipramine was in the top 10 and while animal studies indicate it can improve AD-related deficiencies^88^, the effects on cognition in humans with AD is less clear^89^. Safflower oil in a high fat diet was the 6th highest treatment, but the control in this study was flaxseed oil^90^, so whether the match is more due to one oil over the other is less clear. While D-serine matched as a possible treatment, the datasets for D-serine included an unusually high ratio of upregulated relative to downregulated genes overall^91^ and caution is needed when interpreting that result. The stimulant, cocaine, had three matches in the top 25 and while prolonged use of drugs of abuse induces clear cognitive deficits, the finding is consistent with studies exploring the ability of stimulants to mitigate some aspects of AD, such as apathy^92^. Overall, the ranking of treatments was similar for male and female portrait, although in males, curcumin was the second highest ranked treatment (File 1, Supplementary File 2). 25 individual AD datasets that are brain region specific were compared with each of the treatments and these can be explored in Supplementary File 4. Given that most treatments include only a few regions, comprehensive analysis that involves the same region is still limited. Future studies could focus more on data from specific brain regions (or cell types) from both AD datasets and treatments.

The two treatments that had the lowest treatment scores and were most similar to AD related to alcohol abuse and were from human datasets. Thus, these treatments could be viewed as risk factors for AD. The association of alcohol and AD is complex and still being evaluated^93,94^, but the findings are consistent with work suggesting alcohol abuse as a risk factor for AD^95^.

As a final step, the AD portrait was compared with a recent portrait of depression^14^ as comorbidity of depression and AD occurs in some individuals^96,97^. As shown in Fig. 8, there was a high matching of downregulated genes in both and these included BDNF, CRH, SST, GAD2, and PSEN2. The role of BDNF in depression is actively studied^98,99^ and a connection between exercise and increased BDNF as part of the antidepressant aspects of exercise have been evaluated^100^. In the depression portrait study, exercise also ranked as the top treatment^14^, but the extent of gene reversal was not nearly as large as for AD.

The treatments examined should be viewed as theoretical as the treatment expression studies varied widely across multiple factors, including sex, species (the majority of which were from rodents), numbers, brain region, treatment length, and platform. Most of the datasets were not created with the goal of understanding how the treatment might reverse AD dysregulation patterns and an understanding of experimental design is relevant for interpretation. Also, some recent proposed treatments for AD, such as aducanumab^101^, do not have corresponding large scale gene expression datasets, so they could not be included in this analysis.

We recently used a depression portrait to identify animal models with congruence to depression^102^ and ongoing useful steps could involve use of the AD portrait (or similar portraits) to evaluate and identify what animal model has the highest concordance with the AD brain signature. Advances in this area have already begun as a recent study identified mouse models that were congruent with coexpression modules found in AD^24^.

One goal for producing the AD portrait is to gain new insights into AD but also to produce a platform for identifying and evaluating new treatments at the large-scale gene expression level potential. As shown in Supplementary File 4, a final step involved modifying the AD portrait to include six datasets that came from three studies that were not in the original portrait. The two portraits are extremely similar and ITPKB and GFAP are the top two genes in both. With new datasets and integration approaches any AD portrait will always be ‘in progress’. With evolving portraits and individual datasets, a potentially promising approach is to identify multiple complementary treatments for AD, such as with exercise and fluoxetine.

## Methods

### Creation of gene expression portraits of AD

For the creation of the AD portrait, publicly available large-scale gene expression datasets were used that compared postmortem CNS tissue from humans with AD and controls. Datasets came from both Gene Expression Omnibus (GEO)^103^ and GEO RNA-seq Experiments Interactive navigator (GREIN)^104^. In GEO the datasets were analyzed by the GEO2R tool (for microarray data) and in GREIN the default analysis tool (for RNA-seq data) was used. Both approaches provide information about the two comparison groups (AD vs. control) including p-value, gene symbol, and log of fold change. These files were then converted using R^105^ and RStudio^106^ into files containing two columns: Gene.symbol and sign1, using approaches as described in detail in Ref.^14^. Sign1 indicates degree to which the gene was dysregulated by first − log10 transforming the p value, such that lower p-values have a higher positive value, and then multiplying that value by the sign (positive or negative) of the direction change from control. Therefore, the greater the absolute value of sign1, the greater it was dysregulated. For values that have a negative sign in front of the integer, this indicates a downregulation of that gene in AD. This approach was used to standardize files such that they can be combined to make a portrait, used in making heat maps (see below), and compared with treatments. Each dataset was then updated for proper HUGO symbols.

The portrait was made to identify consistent changes in dysregulated AD genes across multiple brain regions and multiple studies. To accomplish this, a scoring system was used that highlighted the top 1000 up and downregulated genes from each study, but also included information from the top 8000 up and down regulated genes, as described in detail in Ref.^14^. In brief, a ranking system was used whereby genes within the top 1000 and increments of 1000 up to 8000 were assigned a decreasing value. The rationale for using the ranking approach rather than a specific p value is that individual studies vary widely by p value and this approach allows each study to make equal contributions to the portrait. The full script used in R and all dataset text files are available upon request so findings can be reproduced. A maximum of two datasets for each independent study was used so that no one study had an oversized effect on the portrait. Because some studies included males and females and multiple brain regions, these were separately analyzed by sex but including multiple brain regions. These studies included GSE48350, GSE118553, GSE36980, GSE1222063, and GSE5281. For one study, GSE84422, males and females were mixed across analysis platforms, so male and females were analyzed together within a platform. For all other independent studies, two datasets were used. Full information on datasets used for creating the AD portrait and on the individual AD datasets is provided in Supplementary File 1. Together, 22 datasets from 12 independent studies were used to create the AD portrait. Datasets used to create the male and female AD portraits are also provided in Supplementary File 1. After formatting datasets as described above, the portrait was created in RStudio as described^14^.

Using the same datasets to create the AD portrait, an alternative portrait was created using MetaVolcano^26^. The output from MetaVolcano is provided in Supplementary File 3. MetaVolcano includes a focus on specific p values and fold change levels and maintains all genes from all datasets in the final output whereas the portrait created here only includes genes present in more than two thirds of the initial datasets. Finally, a modified AD portrait was made by adding to the original portrait six additional datasets that were harmonized differential expression from the Mayo, ROSMAP, and MSBB studies^31^ (one male and one female for one brain region from each study; see Supplemental File 4 for details). Those datasets were not in the original portrait. The differential expression findings from those studies came from Synapse.org and original source information is provided in Acknowledgements below.

### Comparisons of AD portraits, AD datasets, AD meta-analysis datasets, and a depression portrait

The AD portrait was compared back to each of the 22 datasets from which it was derived to evaluate how accurately it represented the datasets. Further, the AD portrait was compared to each of 67 individual AD datasets. The male and female AD portraits were also compared with the datasets used to create the sex specific portraits. Each AD portrait was compared to one another. Comparisons were performed using a hypergeometric analysis of the top 1000 upregulated and top 1000 downregulated genes between any two datasets. This analysis involved four groups of genes: (A) upregulated genes in both groups, (B) up in first group and down in second, (C) down in first group and up in second group and (D) downregulated in both groups. The hypergeometric derived p-values were − log10 transformed so that lower p-values had a higher value. The scoring system was the same as described in Ref.^14^ and involved adding A and D (same direction), and then subtracting B and C values (opposite direction) to produce a final score. Thus, the higher the overall score, the better the match between two datasets. The outputs of all comparisons with the individual datasets are provided in Supplementary File 1. R studio was used for all analysis. Additionally, the AD portrait was compared with the metaVolcano AD portrait as well as three other recent AD meta-analysis studies^22–24^ (Supplementary File 3). One of the meta-analysis studies^24^ used data from ROSMAP, MSBB, and Mayo and thus had no overlap of starting datasets with the AD portrait. The analysis included both fixed and random approaches and we include results for both. The data from that meta-analysis was downloaded from Synapse and the source is provided in Acknowledgments below. The AD portrait was also compared with a recent portrait of depression in humans^14^. All data from humans used in this study was produced by others, published with assurances of compliance, and made publicly available. For all human summary data from Synapse used in this study, relevant information is provided in the Acknowledgements section below. No new data from humans was produced as part of this study and therefore an accordance statement is not relevant.

### Analysis of the top dysregulated genes

Enrichment analysis for significance (p < 0.05) was conducted using ToppCluster^107^, Enrichr^108^, and STRING^15^ on the top 1000 dysregulated genes. The cutoff of 1000 was used because this is expected to reflect a biological signature and most of the AD datasets have over 1000 significantly dysregulated genes. The reader is encouraged to explore results using different cutoffs. The top 1000 gene list is provided in Supplementary File 1 and can be entered into enrichment tools. Enrichment tests results from ToppCluster and Enrichr (BioPlanet 2019) are provided in Supplementary File 1. The protein–protein interaction tool, STRING, was also used to identify interacting proteins (score of 0.40 or greater) and the to identify genes with high numbers of connections to other genes in the dataset as previously described^14^. Highly interactive genes were either replotted in STRING or exported to Cytoscape^109^ for visualization.

### Analysis of GWAS AD genes with the AD portrait

643 GWAS genes associated with AD from a recent study^25^ were compared with the top 1000 portrait genes. Common genes were identified and hypergeometric overlap test (in R) was performed. A full list of the common AD GWAS and AD portrait genes are provided in Supplementary File 1.

### Potential treatment datasets

201 out of the 252 treatment large-scale gene expression datasets came from a recent study examining potential treatments for depression^14^. Additional treatments that could be relevant for neurodegenerative disorders were added. Using genes from the AD portrait as well as Parkinson’s genes, two drug repurposing tools, NIH LINCS L1000^110^ and Enrichr^108^ were used to identify potential treatments as described in Ref.^14^. GEO datasets^103^ and GREIN^104^ were then queried to identify studies that had been performed using CNS or related tissue (e.g., neuronal stem cells) so that the treatment would have relevance to CNS dysregulation in AD. Only datasets with a minimum of three per group were used. Only datasets with more than 5000 genes in common with the AD portrait were used. Not all potential AD treatments had datasets from the CNS, so these could not be explored further. Any additional treatment that had some evidence for a potential therapeutic effect on AD and that was publicly available was also examined. A majority of the datasets were from mice and rats, but other datasets were from humans, non-human primates, and neuronal tissue, including neuronal cell lines. Creation of the exercise and fluoxetine composites were performed using the same approach as for the AD portraits. The exercise composite included 11 gene expression datasets analyzing the effects of exercise or activity on the brain, including from both human (GSE110298) and rodent (GSE64607, GSE 39697, GSE126996, GSE29075) studies. One study on exercise in SAMP8 transgenic mice (GSE38465) was excluded as it was an outlier compared to other exercise studies. The fluoxetine composite was made from 13 datasets examining the effects of fluoxetine on brain gene expression in mice and rats (GSE28644, GSE84815, GSE118670, GSE43261, GSE48955, and GSE109445). A full list of all treatments tested is shown in Supplemental File 2. The GEO2R tool^103^ was used to obtain a differential gene expression output from microarray data. RNA-seq analysis was performed using GREIN^104^. Together, 252 datasets were used. Each treatment dataset was transformed as were the AD datasets described above whereby the p value is − log10 transformed and multiplied this by the sign of direction of change. The treatment datasets are publicly available and the transformed versions used in this study are available upon request.

### Use of AD portrait, AD datasets, other AD meta-analysis datasets to identify potential treatments

Analysis of the treatments with the AD portraits, other AD meta-analysis studies, and individual AD datasets is identical to that described in detail in Ref.^14^. In brief, a treatment is compared with the AD portrait using a rank rank hypergeometric approach comparing the top 1000 upregulated and top 1000 downregulated genes from each dataset. The potential therapeutic effects were first summed: outputs of B (treatment reversed genes down in AD portrait) and C (treatment reversed genes up in AD). The potential detrimental effects (treatment and disorder in same direction) were then subtracted, namely output of A (treatment upregulates genes already up in AD) and D (treatment downregulates genes down in AD). Thus, a treatment that reversed a high number of AD portrait genes, but had little effect on pushing genes in the same direction would receive a high score. The results of comparison of treatments with the AD portrait, female AD portrait and male AD portrait are provided in Supplementary File 2. Additionally, treatment analysis was performed using the three recent meta-analysis studies, the AD metaVolcano portrait, two AD gene expression studies (separately for males and females) that had the highest number of samples out of all the other datasets (GSE3300 and GSE44771), and 23 other AD datasets; see Supplementary File 4. All analysis was performed using R and script used is available upon request. Heatmap outputs from RRHO^111^ was used to highlight the reversing of AD portrait expression by some of the treatments with portraits and this is useful when examining patterns across all genes.

### Uniform manifold approximation and projection (UMAP) analysis

UMAP^21^ was used as an exploratory approach to gain insights into the performance of some of the top treatments. UMAP incorporates data from complex landscape of multidimensional features (e.g. genes) to identify similarities or differences between datasets. The sign of the AD portrait was reversed so that if a theoretical treatment perfectly reversed all gene expression aspects for the portrait, then it will now match (or be closely aligned with spatially) that portrait. The closer treatments are spatially to the portrait, the better the reversal and potentially better treatment. For the analysis shown, the neighborhood size was set for two different sizes and the correlation clustering tool was used. The top 51 treatments were analyzed. UMAP works well with higher dimensions. Initially the top 8000 AD genes were selected and genes that were missing from the largest number of treatment datasets were removed until 5862 remained. UMAP was run in Python 4.01^112^ using Anaconda (Spyder)^113^.

## Supplementary Information

Supplementary Legends.

Supplementary Information 1.

Supplementary Information 2.

Supplementary Information 3.

Supplementary Information 4.

## Data Availability

All datasets are publicly available datasets or available through Synapse.org as indicated. Datasets were transformed and reformatted as indicated and those versions that were used in this study are available upon request. Script is available upon request to allow for replication of results. Output files are also provided in Supplementary Information. Any additional information can be received by request from SCG.
