# Supplementary material for: Alzheimer’s disease large-scale gene expression portrait identifies exercise as the top theoretical treatment
Source: Sci Rep. 2022 Oct 13;12:17189. doi: 10.1038/s41598-022-22179-z (PMC9561721; doi:10.1038/s41598-022-22179-z)
Supplement: Supplementary file 1 — Supplementary Legends. [file 41598_2022_22179_MOESM1_ESM.docx]

**Supplementary Data**

**Supplementary File 1**

List of Alzheimer’s disease datasets used to make (AD) portraits. Lists of genes in AD portraits. Comparison AD portraits with individual AD datasets. ToppCluster enrichment analysis of top 1000 dysregulated genes in AD portrait. Bioplanet enrichment analysis of top 1000 dysregulated genes in AD portrait. Overlapping genes found in AD GWAS study and AD portrait; top 1000 AD genes that are transcription factors and influence other top AD genes.

**Supplementary File 2**

List of treatments analyzed. Scoring and ranking of treatments that reverse AD expression patterns. Scoring and ranking of treatments that reverse female AD expression patterns. Scoring and ranking of treatments that reverse male AD expression patterns. Lists of genes in exercise composite and genes reversed in AD portrait by exercise in three exercise datasets. ToppCluster enrichment analysis of AD genes reversed by exercise. Bioplanet enrichment of AD genes reversed by exercise.

**Supplementary File 3**

List of metaVolcano AD portrait genes and matching with individual AD datasets. Analysis of matching between multiple AD meta-analysis studies.

**Supplementary File 4**

List of AD datasets, including individual and meta-analysis studies, that are evaluated with all treatments and results.
